# Supplementary material for: COVID-19 serology in nephrology healthcare workers
Source: Wien Klin Wochenschr. 2021 Apr 9;133(17-18):923–30. doi: 10.1007/s00508-021-01848-5 (PMC8034034; doi:10.1007/s00508-021-01848-5)
Supplement: Supplementary file 1 — Supplementary methods, page 3; Supplementary results page 6; Case vignettes of SARS-CoV-2 RT-PCR positive Covid-19 patients, page 8; Figure S1. Points of care at the Division of Nephrology and Dialysis, Department of Medicine III, Medical University of Vienna, Austria, page 9; Figure S2. Disposition of participants, page 10; Figure S3. Nucleocapsid protein immunoblotting for presence of SARS-CoV-2 IgG antibodies in serum, page 11; Figure S4. Spike glycoprotein immunoblotting for presence of SARS-CoV-2 IgG antibodies in serum, page 12; Figure S5. Number of SARS-CoV-2 antibody positive subjects at follow-up by profession, page 13; Figure S6. Number of Covid-19 cases in Austria, page 14; Figure S7. Containment measures, mandated by the federal government of Austria, page 15; Figure S8. Containment measures in Vienna and at the Medical University of Vienna, page 16; Table S1. Details of commercial laboratory tests used for follow-up confirmation of all 60 participants who tested borderline positive or positive for SARS-CoV-2 antibodies at baseline page 17; Table S2. Detailed IgM and IgA antibody test results at baseline and follow-up of all 18 subjects with a positive antibody test at follow-up, and of five Covid-19 patients, page 18; Table S3. Detailed IgG antibody and total antibody test results at baseline and at follow-up of 18 subjects with a positive antibody test at follow-up, and of five Covid-19 patients, page 19. [file 508_2021_1848_MOESM1_ESM.pdf]

Supplementary material

COVID-19 serology in nephrology health care workers

Table of contents

Supplementary methods 3

Supplementary results **6**

Case vignettes. SARS-CoV-2 RT-PCR positive Covid-19 patients. 8

Figure S1. Points of care at the Division of Nephrology and Dialysis, Department of Medicine III, Medical University of Vienna, Austria. 9

Figure S2. Disposition of participants. 10

Figure S3. Nucleocapsid protein immunoblotting for presence of SARS-CoV-2 IgG antibodies in serum. 11

Figure S4. Spike glycoprotein immunoblotting for presence of SARS-CoV-2 IgG antibodies in serum. 12

Figure S5. Number of SARS-CoV-2 antibody positive subjects at follow-up by profession 13

Figure S6. Number of Covid-19 cases in Austria. 14

Figure S7. Containment measures, mandated by the federal government of Austria. 15

Figure S8. Containment measures in Vienna and at the Medical University of Vienna. 16

Table S1. Details of commercial laboratory tests used for follow-up confirmation of all 60 participants who tested borderline positive or positive for SARS-CoV-2 antibodies at baseline. 17

Table S2. Detailed IgM and IgA antibody test results at baseline and follow-up of all 18 subjects with a positive antibody test at follow-up, and of five Covid-19 patients. 18

Table S3. Detailed IgG antibody and total antibody test results at baseline and at follow-up of 18 subjects with a positive antibody test at follow-up, and of five Covid-19 patients. 19

Supplementary methods

Commercial SARS-CoV-2 antibody tests

We used the following enzyme-linked immunosorbent assays (ELISA), chemiluminescence immunoassay (CLIA), and electrochemiluminescence immunoassay (ECLIA), for all follow-up analyses: SARS-CoV-2 nucleocapsid protein (NP) ELISA, IgG class antibodies (ImmunoDiagnostics, Hongkong); SARS-CoV-2 nucleocapsid protein (NP) ELISA, IgM class antibodies (ImmunoDiagnostics, Hongkong); EDI Novel Coronavirus COVID-19 IgG ELISA kit (Epitope Diagnostics Inc., San Diego, CA); EDI Novel Coronavirus COVID-19 IgM ELISA kit (Epitope Diagnostics Inc., San Diego, CA); Anti-SARS-CoV-2-ELISA IgG (Euroimmun Medizinische Labordiagnostika AG, Lübeck, Germany); Anti-SARS-CoV-2-ELISA IgA (Euroimmun Medizinische Labordiagnostika AG, Lübeck, Germany); Wantai SARS-CoV-2 total antibody ELISA (Beijing Wantai Biological Pharmacy Enterprise Co., Ltd., Beijing, China); Wantai SARS-CoV-2 IgM ELISA (Beijing Wantai Biological Pharmacy Enterprise Co., Ltd., Beijing, China); Liaison SARS-CoV-2 S1/S2 IgG CLIA (DiaSorin S.p.A, Saluggia, Italy); Elecsys Anti-SARS-CoV-2 ECLIA (Roche Diagnostics Deutschland G.m.b.H, Mannheim, Germany).Technical details of these tests are indicated in **Supplementary Table S1.**

In subjects with IgG antibodies at follow-up and for Covid-19 control samples we also used two additional ELISAs: COVID-19 ELISA IgG testkit (Vircell, Granada, Spain; nucleocapsid protein and spike glycoprotein antigen) and the ID Screen ® SARS-CoV-2-N IgG Indirect ELISA (IDvet, Grabels, France; nucleocapsid protein antigen).

Western blotting

One µg of the SARS-CoV-2 virus nuclear capsid protein purchased from Prospec-Tany Technogene Ltd. (Ness-Ziona, Israel) or of the SARS-CoV-2 spike glycoprotein was loaded onto a flat comb SDS-PAGE gel (BioRad Hercules, CA) and run under reducing conditions using tris-glycine-SDS buffer. The gel was blotted by semi-dry blotting on nitrocellulose. After blocking the filter with Roche blocking buffer (Merck KGaA, Darmstadt, Germany) at room temperature for 30 minutes, the filter was put into the MilliBlot-MP membrane processor (Merck KGaA, Darmstadt, Germany) and 230µl of patient serum diluted 1:100 was applied to each lane. Incubation was performed at room temperature (2.5 h) with continuous shaking. After two short washes with TPBS through the injection port of the MilliBlot device, the filter was removed into an incubation tray and washed further with TPBS (2x10 min). To detect the binding of SARS-CoV-2 nuclear capsid protein-specific IgG antibodies, the filter

was incubated with HRP-conjugated anti-human IgG immunoglobulin (1:4000 in PBS) for 60 min at room temperature. After two washing steps of 10 min each with TPBS, the signal was developed with Roche chemiluminescent reagent (Merck KGaA, Darmstadt, Germany) and recorded with the Fusion FX Vilber Lourmat (Vilber, Eberhardzell, Germany). Images were further processed with Adobe Photoshop version 6 (Adobe, San Jose, CA).

SARS-CoV-2 neutralization assay

The virus used for the serum neutralization assay was passaged and expanded twice before titration by $TCID_{50}$ on Vero 76 clone E6 cells (CCLV-RIE929, Friedrich-Loeffler-Institute, Riems, Germany) after its initial isolation from a clinical sample (nasopharyngeal swab, sampled in mid-March 2020 from a 25-year old male patient in Lower Austria; the isolate was submitted for next generation sequencing, full length sequence pending). The neutralization assay was set up in flat-bottom 96-well tissue culture plates. Human sera were heat-treated for 30 min at 56°C and 1 in 10 diluted in serum-free HEPES medium (Cell culture service, AGES, Mödling, Austria) as starting point for the assay. The two-fold serially diluted sera were incubated with an equal volume of 50 µl SARS-CoV-2 at a minimum of 2000 TCID50/ml for 90 min at 37°C. Afterwards, 25,000 Vero 76 clone E6 cells were added to each well to the serum/virus mixture in a volume of 100 µl in Eagle's minimum essential medium supplemented with 10% FBS and incubated for 4 days at 37°C, 5% CO_2 in a humidified incubator. The cytopathic effect (CPE) in every well was scored under an inverted optical microscope and the reciprocal of the highest serum dilution that protected more than 50% of cells from CPE was taken as the neutralizing titer.

Detection of SARS-CoV-2

We used the Roche Cobas® SARS-CoV-2 PCR test on the Roche® Cobas 6800 platform (Roche Diagnostics Deutschland GmbH, Mannheim, Germany) for the detection of SARS-CoV-2 RNA in nasopharyngeal or oropharyngeal swab specimens. Tests were done individually or as pool-tests (up to ten samples per analysis).

Definition of SARS-CoV-2 contact category 1 and category 2 individuals

A category 1 person is defined as an individual who has high risk of exposure to SARS-CoV-2 (such as living with a Covid-19 patient, direct contact to a Covid-19 patient), whereas category 2 person refers to an individual who has a low risk of exposure to SARS-CoV-2 (such as being in a room with a Covid-19 positive patient for a certain amount of time within a specific distance).

Supplementary results

Commercial antibody tests

SARS-CoV-2 antibodies – *follow-up negatives*

At follow-up, 28 of 60 initially borderline positive or positive participants tested negative with all ten commercial laboratory tests and Western blots for nucleocapsid protein and spike glycoprotein. Of the 28 negatives at follow-up, results at baseline using the Immunodiagnostics ELISAs, were as follows: four tested borderline positive and 20 positive for IgM, two borderline positive for IgG, one combined borderline positive for IgM and IgG, and 1 combined borderline positive for IgM and positive for IgG, **Figure 1**.

SARS-CoV-2 antibodies – *follow-up borderline positives*

Fourteen participants showed borderline positive results in one or two of the ten commercial tests and were negative by anti SARS-CoV-2 nucleocapsid protein and spike glycoprotein IgG Western blots at follow-up: seven IgM, ImmunoDiagnostics; five IgA, Euroimmun; one IgG, Liaison; one IgM, ImmunoDiagnostics and IgA, Euroimmun. With regard to the Immunodiagnostics ELISA results at baseline, out of 10 participants who were borderline positive for IgM at baseline, five remained borderline positive and five were negative for IgM at follow-up. Three of four IgM positive individuals at baseline were borderline positive for IgM at follow-up and one was negative at follow-up.

SARS-CoV-2 antibodies - *follow-up positives*

Among 18 antibody positive subjects, as compared to the baseline results of the ImmunoDiagnostics ELISAs, four out of eight initially IgM positive subjects remained positive, three were borderline positive, and one was negative at follow up. Four IgM borderline positive individuals were also negative at follow-up. The six others comprised three IgG borderline and three IgG positive subjects who showed mixed results by the ImmunoDiagnostics ELISAs at follow-up (**Figure 2** and **Supplementary Table S2** and **S3**). None of these individuals showed a relevant increase in IgM or IgG antibodies by ImmunoDiagnostics ELISAs at follow-up.

Among these 18 individuals, 10 were tested negative by the anti SARS-CoV-2 nucleocapsid protein IgG Western blot (four showed IgM by ImmunoDiagnostics, one IgM by Wantai, one IgA by Euroimmun, three IgG (one Epitope Diagnostics, two Liaison), and one total antibody by Roche). Among eight subjects tested positive by nucleocapsid protein Western blot (**Supplementary Figure S3**), two showed no other positive result (one had borderline

positive IgM by ImmunoDiagnostics); two were positive for IgG by Epitope Diagnostics (one of them borderline positive for IgM by ImmunoDiagnostics); one showed IgM by ImmunoDiagnostics and by Wantai and was borderline positive for IgG by ImmunoDiagnostics; three had IgG by ImmunoDiagnostics (one of them had also IgA by Euroimmun and another one was borderline positive for IgG by Epitope Diagnostics). Of note, none of these 18 persons judged to be positive in one of the commercial tests or IgG nucleocapsid protein Western blot had IgG by Euroimmun, total antibody by Wantai, or spike glycoprotein Western blot (**Supplementary Figure S4**), or a clear positive total antibody result by Roche.

Among 12 IgG positive individuals four had a negative nucleocapsid protein Western blot, but two of them tested positive for anti-nucleocapsid protein IgG (Epitope Diagnostics) or total antibody (Roche) and two for anti-spike glycoprotein IgG (Liaison). Seven nucleocapsid protein Western blot positive individuals had anti-nucleocapsid IgG (two borderline positive by ImmunoDiagnostics, three positive by ImmunoDiagnostics (one of them borderline positive for IgG by Epitope Diagnostics), two were positive for IgG by Epitope Diagnostics). One participant tested positive only by nucleocapsid protein Western blot.

SARS-CoV-2 antibodies – *Covid-19 positive controls*

All five patients tested positive for anti-nucleocapsid protein IgG by Western blot and two of them positive for anti-spike glycoprotein IgG by Western blot. One showed total antibody by Roche and was borderline positive for IgG by ImmunoDiagnostics, two tested positive with all commercial tests, one tested positive for all commercial tests except IgM by ImmunoDiagnostics and Epitope Diagnostics, and was only borderline positive for IgG by ImmunoDiagnostics, and the last one was positive with all tests except IgM by Epitope Diagnostics and was borderline positive for IgM by ImmunoDiagnostics and borderline positive for IgG by Liaison. All five patients also showed IgG by the Vircell and the IDvet ELISAs (**Figure 2**, **Supplementary Table S2** and **S3**).

Case vignettes. SARS-CoV-2 RT-PCR positive Covid-19 patients

C1: A 32-year old male patient was treated with high dose oral steroid due to severe nephrotic syndrome caused by a biopsy-proven minimal change disease in early March 2020. At this time contact tracing of another person who tested positive for SARS-CoV-2 resulted in home isolation of our patient, who also tested positive for SARS-CoV-2 by RT-PCR of a nasopharyngeal swab specimen. He developed only mild gastro-intestinal symptoms typical for Covid-19 and achieved complete remission of his nephrotic syndrome within four weeks. At this point in time, viral RNA was no longer detectable, and he tested positive for SARS-CoV-2 antibodies.

C2: A 43-year old male took a skiing holiday in Tyrol at the beginning of March 2020. One week following his return to Vienna he had no signs of upper respiratory tract infection or pneumonia, but joint pain. Subsequently a positive SARS-CoV-2 RT-PCR confirmed very mild Covid-19. In a blood sample taken about five weeks later, he tested positive for SARS-CoV-2 antibodies.

C3: A 44-year old female developed severe Covid-19 presenting with severe respiratory distress syndrome and requiring treatment at an intensive care unit. A serum sample, positive for SARS-CoV-2 antibodies, was taken about 8 weeks after her first positive SARS-CoV-2 RT-PCR in March 2020.

C4: A 60-year-old female presented with mild to moderate Covid-19, resulting in weakness, fever and respiratory symptoms, confirmed by RT-PCR, at the end of March. She recovered after 3 weeks of illness and had a positive SARS-CoV-2 antibody test at the end of 4 weeks of home isolation after start of symptoms.

C5: A 61 year old male suffered from RT-PCR proven mild Covid-19 with fatigue and respiratory infection at the end of March and recovered rapidly within two weeks of home isolation. He tested positive for SARS-CoV-2 antibodies in a blood sample taken about 4 weeks after onset of symptoms.

Supplementary Figures and Tables.

Figure S1. Points of care at the Division of Nephrology and Dialysis, Department of Medicine III, Medical University of Vienna, Austria.

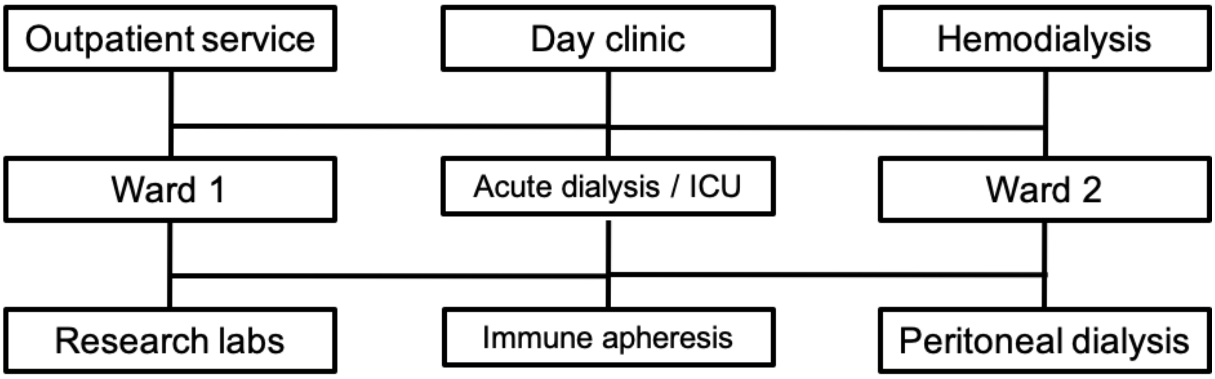

Figure S2. Disposition of participants (admin denotes administrative staff).

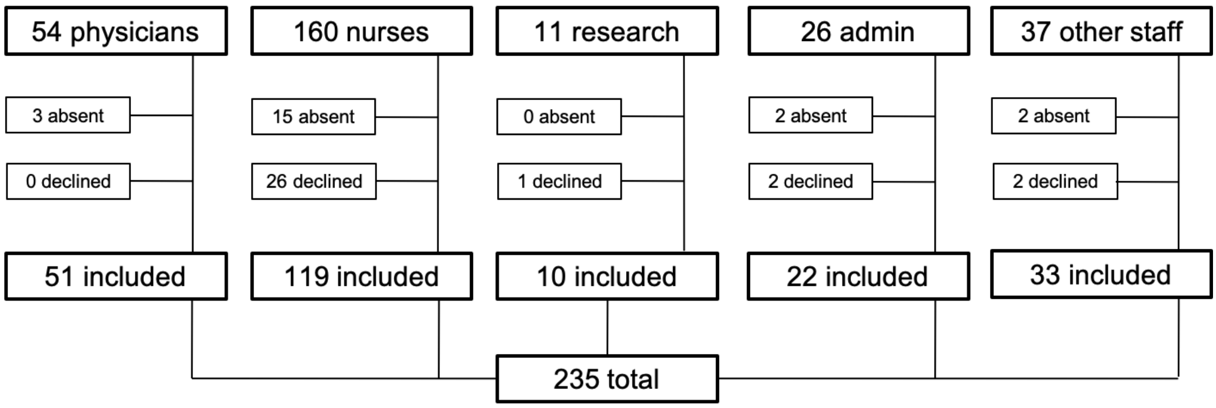

Figure S3. Nucleocapsid protein immunoblotting for presence of SARS-CoV-2 IgG antibodies in serum.

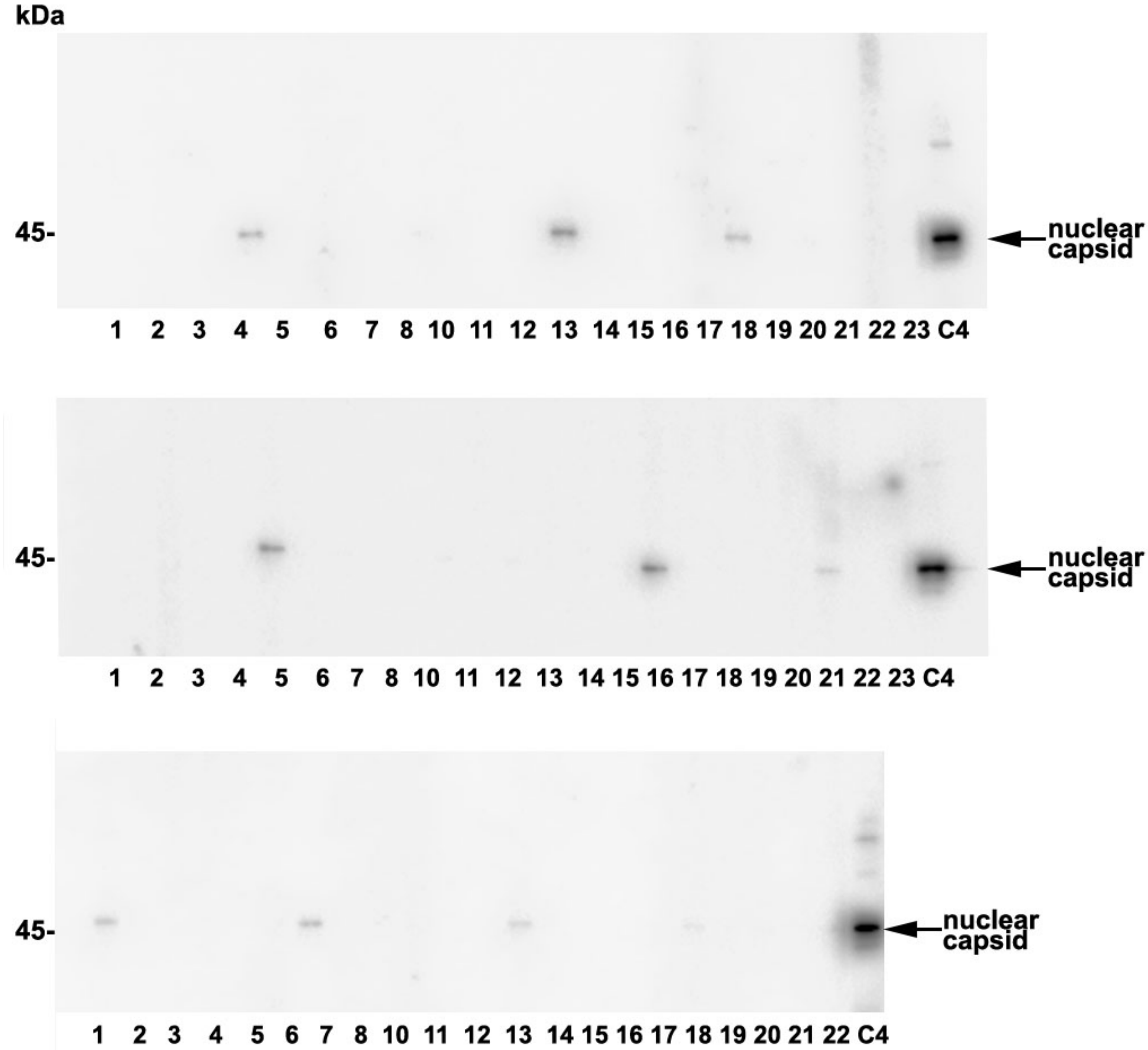

Nucleocapsid protein immunoblotting for presence of anti SARS-Cov-2 IgG antibodies in serum.

One µg of original purified SARS-Cov-2 nucleocapsid protein was loaded onto a 12% IPG/prep-well comb gel (Bio-Rad #456-1041). The resultant PAGE gel was blotted onto nitrocellulose membrane, which was then inserted into the membrane processor (Milliblot-MP; Millipore, Bedford, MA, USA) and 230 µL of 1:100 diluted donor serum was applied into each slot. After incubation of 180 min at RT the entire membrane was removed from the membrane processor and incubated for two washing steps for 10 min each with TPBS. Finally, the membrane was exposed to the HRP-conjugated antibody detection solution for 60 min at RT and developed using chemiluminescence reagent. Upper panel #4, 13, 18, C4 represent #9, 200, 222, C4 at the HEATMAP (Fig 2). Middle panel #5, 16, 21, C4 represent #127, 133, 148, C4 at the HEATMAP (Fig 2). Lower panel #1, 13, C4 represent #65, 67, C4

at the HEATMAP (Fig 2). Number 7 at lower panel depicts patient #127 at the HEATMAP two weeks earlier. Blots, which were processed on different days were clearly separated from others by a white space. The arrow indicates the recombinant nucleocapsid protein as obtained from Prospec-Tany Technogene Ltd. (Ness-Ziona, Israel).

Figure S4. Spike glycoprotein immunoblotting for presence of SARS-CoV-2 IgG antibodies in serum.

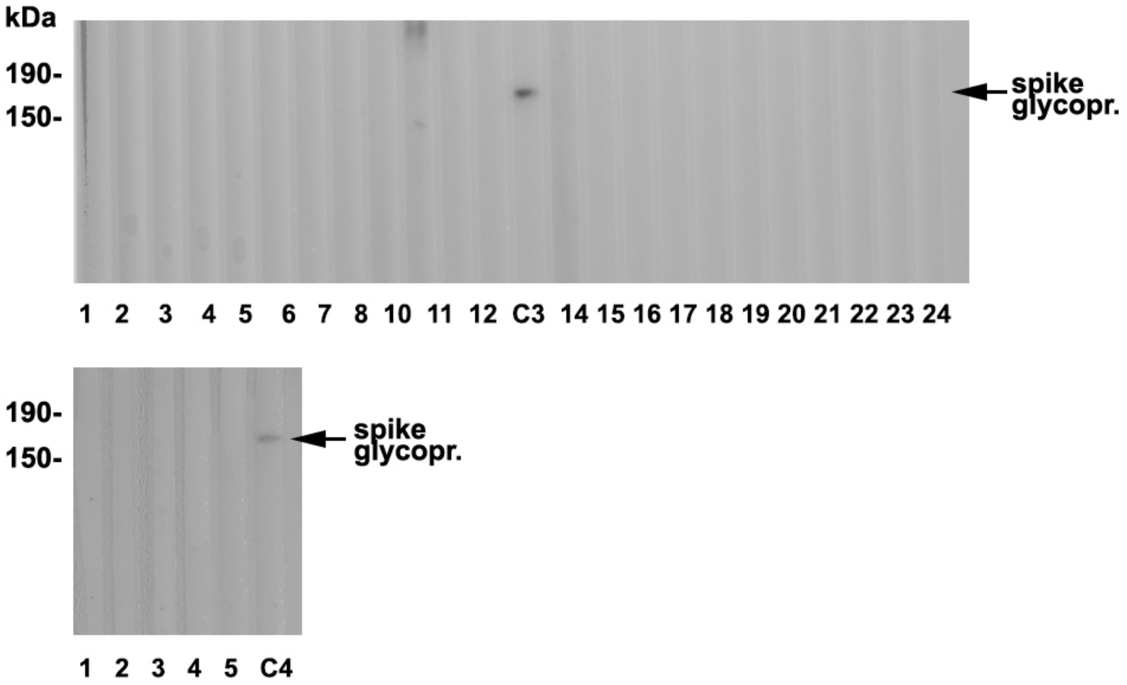

Spike protein immunoblotting for presence of anti SARS-Cov-2 IgG antibodies in serum.

One and a half µg of original purified spike surface glycoprotein (as indicated in Amanat F, Stadlbauer D, et al. Nature Med., 2020) was loaded onto a 12% IPG/prep-well comb gel (Bio-Rad #456-1041). The resultant PAGE gel was blotted onto nitrocellulose membrane, which was then inserted into the membrane processor (Milliblot-MP; Millipore, Bedford, MA, USA) and 230 µL of 1:100 diluted donor serum was applied into each slot. After incubation of 180 min at RT the entire membrane was removed from the membrane processor and incubated for two washing steps for 10 min each with TPBS. Finally, the membrane was exposed to the HRP-conjugated antibody detection solution for 60 min at RT and developed using chemiluminescence reagent. Upper panel #13 (termed C3) represents C3 at the HEATMAP (Fig 2). Lower panel #23 (termed C4) represents C4 at the HEATMAP (Fig 2). The arrow indicates the spike surface glycoprotein as obtained (Amanat F. et al., 2020). Molecular size markers are presented to the left. Blots, which were processed on different days were clearly separated from others by a white space.

Figure S5. Number of SARS-CoV-2 antibody positive subjects at follow-up by profession.

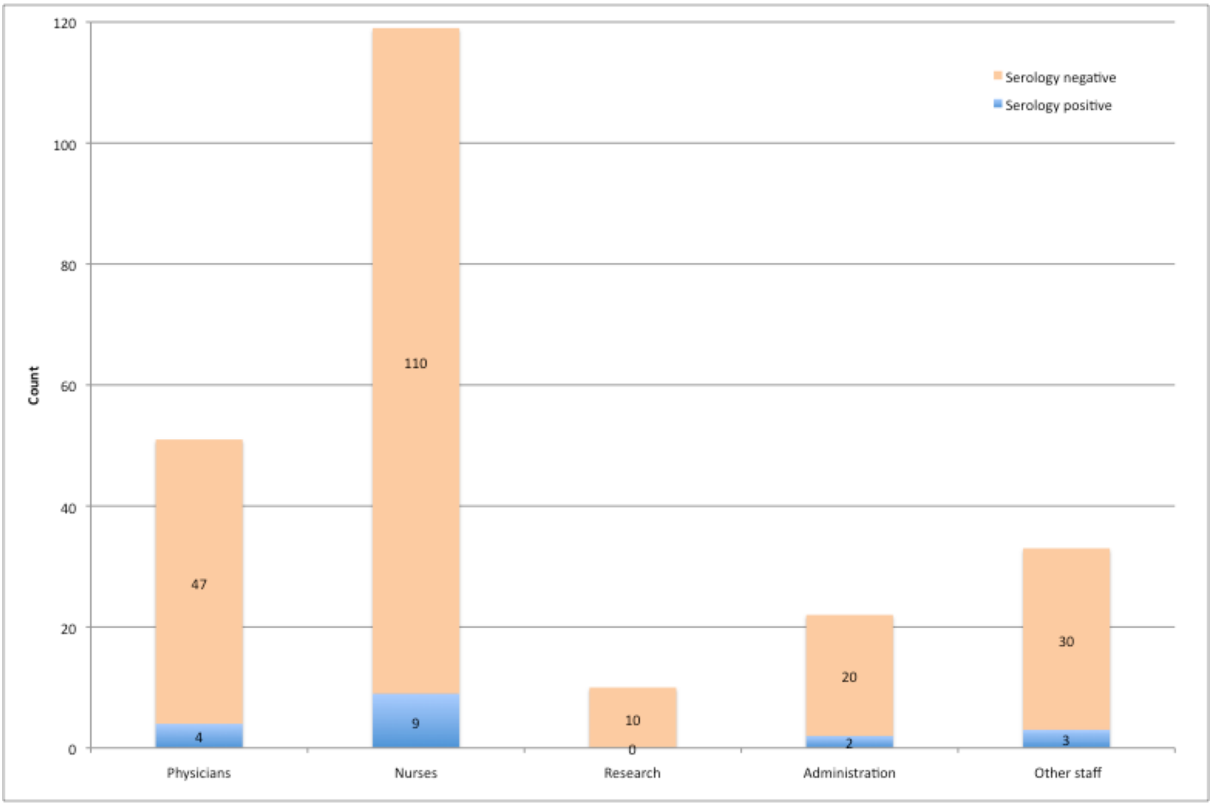

Figure S6. Number of Covid-19 cases in Austria.

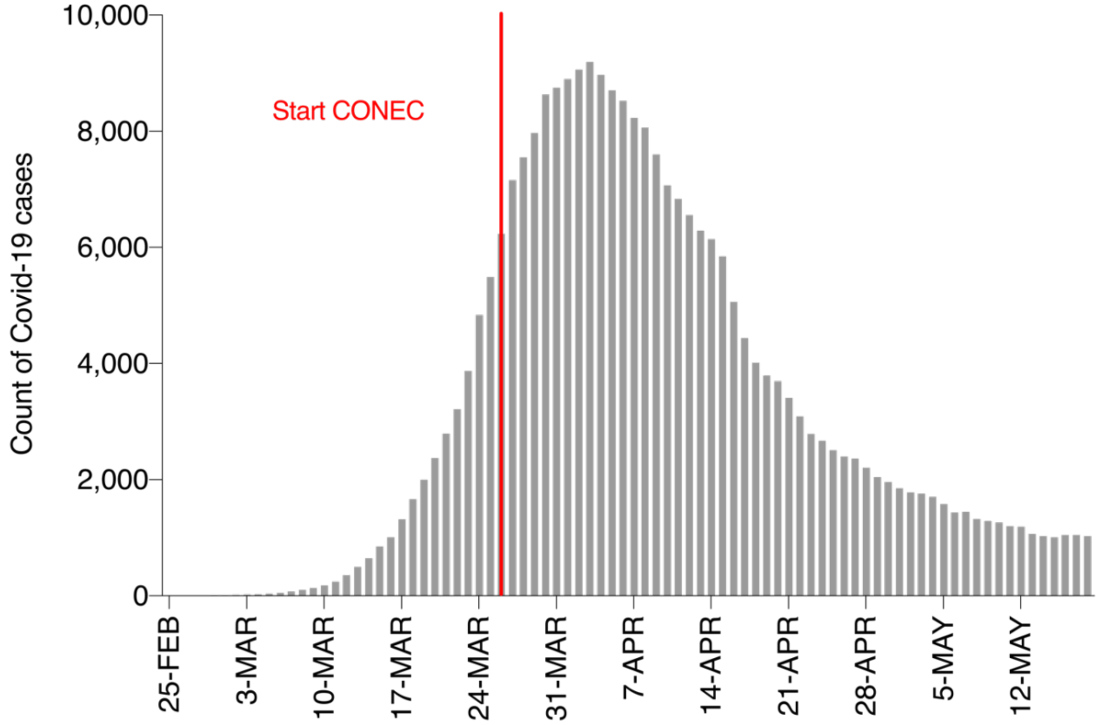

The first two patients tested positive for SARS-CoV-2 on February 25th 2020 in Innsbruck, Tyrol, reaching a maximum of 9.193 cases on April 3rd 2020.

Data provided by the official Covid-19 dashboard of the Austrian Department of Health ("Bundesministerium für Soziales, Gesundheit, Pflege und Konsumentenschutz"), https://info.gesundheitsministerium.at/?l=en (accessed May 21st 2020)

Figure S7. Containment measures, mandated by the federal government of Austria.

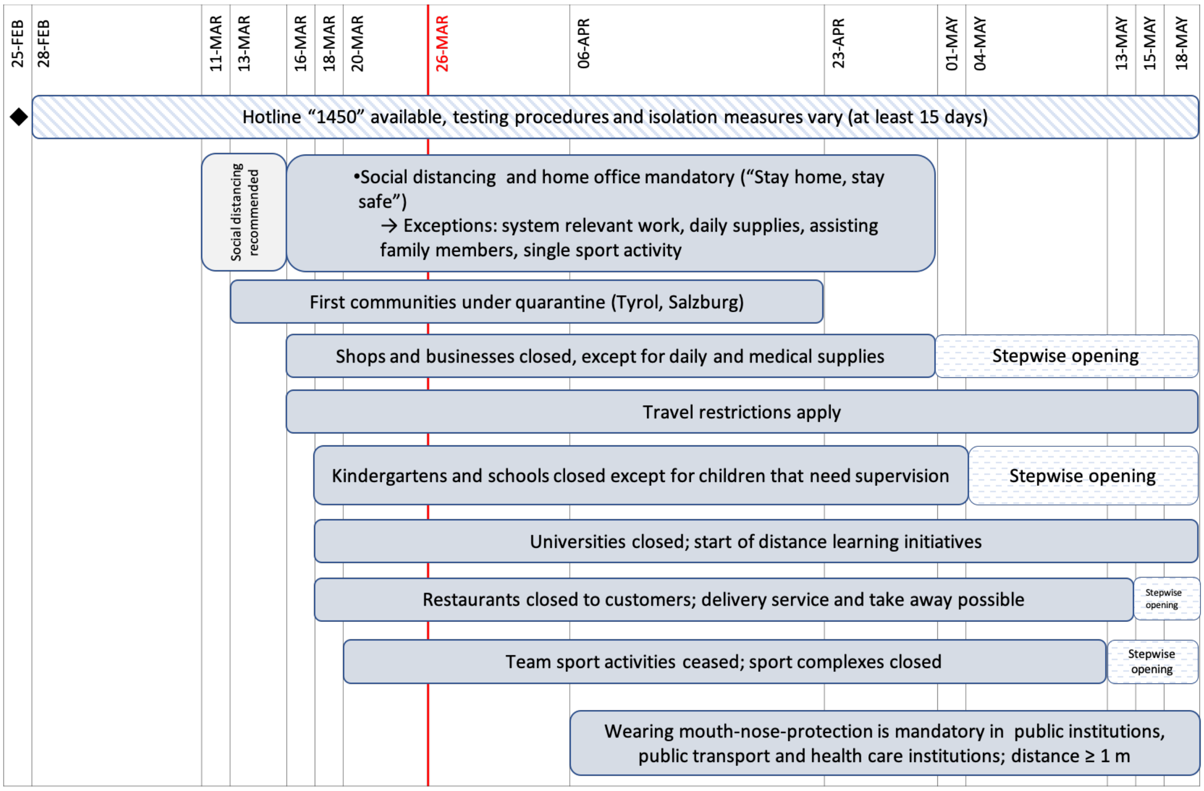

◆ First infections registered in Austria.

Beginning with recommendations for social distancing in the first days of March, measures issued by the Federal government were quickly escalated to a thorough lockdown of public life.

The red line indicates the start of the CONEC study.

Figure S8. Containment measures in Vienna and at the Medical University of Vienna.

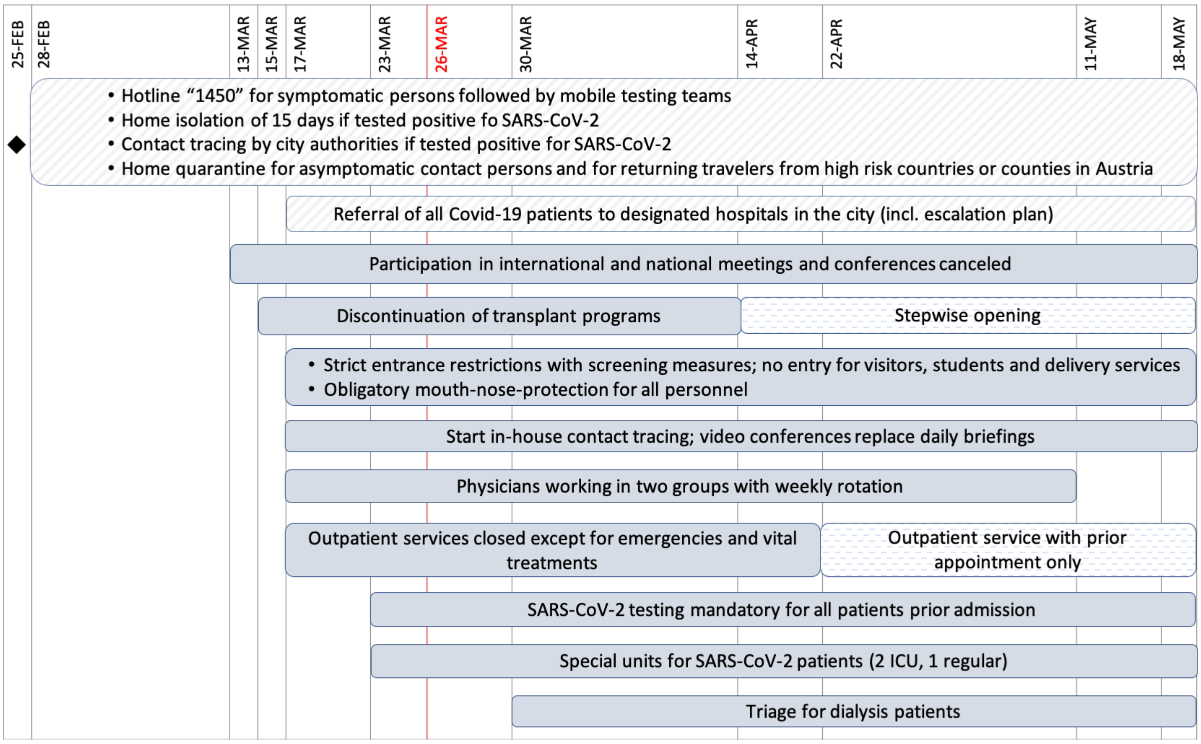

◆ First infections registered in Austria.

Vienna city authorities extended measures taken by the federal government in Austria and issued an escalation plan for adequate medical care (marked striped). Designated hospitals were primarily responsible for medical care of Covid-19 patients while others were intended to be Covid-19 free.

In addition, the Medical University of Vienna took several steps to ensure the readiness of the medical staff and to designate Covid-19 patients as early as possible to specialized centers (marked in grey).

The red line indicates the start of the CONEC study.

Table S1. Details of commercial laboratory tests used for follow-up confirmation of all 60 participants who tested borderline positive or positive for SARS-CoV-2 antibodies at baseline.

Company	IMD	EDI	EUR	LIA	ROC	WAN	IMD	EDI	WAN	EUR
Method	ELISA	ELISA	ELISA	CLIA	ECLIA	ELISA	ELISA	ELISA	ELISA	ELISA
Antibody	IgG	IgG	IgG	IgG	TA	TA	IgM	IgM	IgM	IgA
Epitope	N	N	S_1	S_1 & S_2	N	S_1	N	N	S_1	S_1
Material	serum / plasma	serum	serum / plasma	serum / plasma	serum / plasma	serum / plasma	serum / plasma	serum	serum / plasma	serum / plasma
Sample dilution	1:100	1:100	1:101	1:10	-	100 µl	1:100	1:10	1:10	1:101
Incubation	60 min at RT	30 min at RT	60 min at 37°C	n.a.	18 min	30 min at 37°C	60 min at RT	30 min at 37°C	30 min at 37°C	60 min at 37%
Result interpretation	OD	OD and CO	ratio	AU/ml	COI	A/CO	OD	OD and CO	A/CO	ratio
Negative	<0.200	<neg CO	<0.8	<12	<1.0	≤0.9	<0.200	<neg CO	≤0.9	<0.8
Borderline	0.200 - <0.300	neg CO – pos CO	≥0.8 - <1.1	12 - <15	-	0.91 - <1.1	0.200 - <0.300	neg CO – pos CO	0.91 - <1.1	≥0.8 - <1.1
Positive	≥0.300	>pos CO	≥1.1	≥15	≥1.0	≥1.1	≥0.300	>pos CO	≥1.1	≥1.1
CV, intra assay	4.93% - 5.68%	<15%	3.9% - 16%	2.6% - 5.3%	-	-	4.93% - 6.77%	<15%	-	2.4% - 13.7%
CV, inter assay	5.46% - 6.75%	<20%	0%- 16.2%	0.5% - 11%	-	-	5.35% - 7.16%	<20%	-	0% - 16.1%
Specificity	93.89%	100%	98.50%	98.50%	99.81%	100%	-	97.70%	100%	92.50%
Sensitivity	93.33%	100%	33.3% - 80%	25% - 97.4%	65.5% - 100%	94.50%	-	45%	86.80%	50% -100%

IMD, ImmunoDiagnostics, Hongkong; EDI, Epitope Diagnostics Inc., San Diego, CA; EUR, Euroimmun Medizinische Labordiagnostika AG, Lübeck, Germany; WAN, Beijing Wantai Biological Pharmacy Enterprise Co., Ltd., Beijing, China; LIA, Liaison, DiaSorin S.p.A, Saluggia, Italy; ROC, Roche Diagnostics Deutschland G.m.b.H, Mannheim, Germany

ELISA, enzyme-linked immunosorbent assay; CLIA, chemiluminescence immuno assay; ECLIA, electrochemiluminescence immunoassay

TA, total antibody; N, SARS-CoV-2 nucleocapsid protein; S_1, SARS-CoV-2 spike glycoprotein S_1 subunit; S_2, SARS-CoV-2 spike glycoprotein S_2 subunit

RT, room temperature; OD, optical density; AU/ml, arbitrary units/ml; COI, cutoff index; A/CO, sample extinction/cutoff; CV, coefficient of variation

EUR-IgG & -IgA: Ratio = (extinction sample/extinction calibrator), ratio < 0.8 = negative, ratio <1.1 = borderline, ratio ≥1.1 = positive

WAN-TA & -IgM: Ratio = (extinction sample/cutoff), ratio ≤ 0.9 = negative, ratio 0.91- <1.1 = borderline, ratio ≥1.1 = positive

EDI-IgG: positive = > 1.1 x (negative control + 0.18), negative = < 0.9 x (negative control + 0.18)

EDI-IgM: positive = > 1.1 x (negative control + 0.10), negative = < 0.9 x (negative control + 0.10)

LIA-IgG: AU/ml <12 =negative, 12-<15 = borderline, ≥ 15= positive

ROC: cutoff index = (sample signal/cutoff), < 1.0 = negative, ≥ 1.0 = positive

IMD-IgG & -IgM: OD < 0.200 = negative, OD ≥ 0.300 = positive

Table S2. Detailed IgM and IgA antibody test results at baseline and follow-up of all 18 subjects with a positive antibody test at follow-up, and of five Covid-19 patients.

Company	IMD	IMD	IMD	IMD	EDI	EDI	WAN	WAN	EUR	EUR
Ig	IgM	IgM	IgM	IgM	IgM	IgM	IgM	IgM	IgA	IgA
Antigen*	N-1	N-2	N-1	N-2	N-1	N-2	S-1	S-2	S-1	S-2
Time	BL	BL	FU	FU	FU	FU	FU	FU	FU	FU
ID										
36	0.244	0.308	0.442	0.348	0.132	0.125	0.002		0.108	0.106
76	0.537	0.530	0.459	0.460	0.126	0.152	0.005		0.160	0.208
165	0.856	0.513	0.331	0.316	0.087	0.120	0.056		0.267	0.192
120	0.494	0.493	0.200	0.225	0.129	0.117	0.178	0.224	0.114	0.113
179	0.497	0.474	0.303	0.298	0.136	0.121	0.004		0.304	0.279
197	0.224	0.149	0.078	0.076	0.114	0.100	0.006		0.423	0.412
92	0.202	0.207	0.183	0.172	0.138	0.156	0.007		0.204	0.150
123	0.398	0.317	0.210	0.215	0.107	0.134	0.006		0.248	0.280
167	0.200	0.154	0.107	0.108	0.095	0.128	0.015		0.186	0.125
205	0.328	0.217	0.162	0.157	0.101	0.121	0.012		0.287	0.321
9	0.155	0.159	0.312	0.074	0.098	0.092	0.502	0.582	0.134	0.130
67	0.389	0.383	0.239	0.253	0.101	0.103	0.100		0.149	0.150
133	0.410	0.410	0.184	0.224	0.135	0.129	0.002		0.169	0.167
222	0.292	0.303	0.195	0.192	0.126	0.132	0.023		0.112	0.117
200	0.248	0.261	0.157	0.161	0.097	0.099	0.006		0.622	0.632
65	0.237	0.233	0.128	0.129	0.116	0.124	0.004		0.165	0.156
127	0.178	0.182	0.163	0.166	0.111	0.133	0.002		0.209	0.247
148	0.217	0.159	0.094	0.091	0.126	0.114	0.006		0.145	0.137
C1			0.298	0.294	0.135	0.149	0.678		0.720	0.735
C2			0.090	0.090	0.103	0.124	0.055		0.172	0.178
C3			0.136	0.116	0.109	0.106	3.024		OVRFLW	OVRFLW
C4			2.117	2.239	0.487	0.566	3.500		2.607	2.685
C5			1.136	1.350	0.491	0.490	1.114		0.739	0.698

IMD ImmunoDiagnostics, HongKong; EDI, Epitope Diagnostics Inc., San Diego, CA; WAN, Beijing Wantai Biological Pharmacy Enterprise Co., Ltd., Beijing, China; EUR, Euroimmun Medizinische Labordiagnostika AG, Lübeck, Germany; N, SARS-CoV-2 nucleocapsid protein; S, SARS-CoV-2 spike glycoprotein; BL, baseline; FU, follow-up; neg, negative; pos, positive; OVRFLW, overflow.

*, numbers 1 and 2 indicate raw data of duplicates.

Table S3. Detailed IgG antibody and total antibody test results at baseline and at follow-up of 18 subjects with a positive antibody test at follow-up, and of five Covid-19 patients.

Company	IMD PRNT	IMD	IMD	IMD	EDI	EDI	VIR	VIR	IDV	IDV	WB	EUR	EUR	LIA	LIA	WB	ROC	ROC	WAN	WAN	
Ig	IgG	IgG	IgG	IgG	IgG	IgG	IgG	IgG	IgG	IgG	IgG	IgG	IgG	IgG	IgG	IgG	TA	TA	TA	TA	
Antigen*	N-1	N-2	N-1	N-2	N-1	N-2	NS-1	NS-2	N-1	N-2	neg	S-1	S-2	S-1	S-2	S	N-1	N-2	S-1	S-2	
Time	BL	BL	FU	FU	FU	FU	FU	FU	FU	FU	FU	FU	FU	FU	FU	FU	FU	FU	FU	FU	FU
ID																					
36	0.081	0.090	0.099	0.062	0.192	0.181					neg	0.044	0.047	6.45		neg	0.075		0.001		
76	0.073	0.066	0.063	0.071	0.193	0.220					neg	0.132	0.131	5.76		neg	0.080		0.003		
165	0.075	0.057	0.058	0.060	0.277	0.303					neg	0.069	0.060	<3,8		neg	0.085		0.003		
120	0.076	0.069	0.061	0.054	0.214	0.228					neg	0.061	0.057	<3,8		neg	0.073		0.005	0.001	
179	0.077	0.065	0.061	0.059	0.195	0.210					neg	0.128	0.126	3.85		neg	0.086		0.004		
197	0.097	0.063	0.068	0.067	0.221	0.228					neg	0.057	0.056	<3,8		neg	0.084		0.003		
92	0.088	0.058	0.053	0.053	0.973	0.707	0.179	0.181	0.063	0.076	neg	0.054	0.050	<3,8		neg	0.080		0.006		<10
123	0.224	0.158	0.183	0.195	0.205	0.243	0.205	0.217	0.048	0.052	neg	0.072	0.067	<3,8	3.86	neg	1.040	1.040	0.004		<10
167	0.091	0.059	0.059	0.062	0.150	0.164	0.182	0.190	0.060	0.058	neg	0.075	0.056	27.70	27.70	neg	0.294	0.304	0.070		<10
205	0.063	0.049	0.053	0.055	0.145	0.163	0.222	0.214	0.051	0.061	neg	0.052	0.052	79.10	79.10	neg	0.084	0.086	0.003		<10
9	0.250	0.258	0.286	0.075	0.238	0.228	0.312	0.317	0.062	0.059	pos	0.054	0.047	<3,8		neg	0.092		0.005	0.008	<10
67	0.160	0.158	0.138	0.137	0.649	0.641	0.276	0.248	0.075	0.076	pos	0.097	0.098	<3,8	4.40	neg	0.084	0.087	0.004		<10
133	0.087	0.083	0.074	0.068	0.748	0.704	1.892	1.870	1.103	1.102	pos	0.083	0.086	<3,8	<3,8	neg	0.080	0.078	0.003		<10
222	0.297	0.223	0.300	0.305	0.232	0.230	0.231	0.233	0.096	0.116	pos	0.093	0.097	5.50		neg	0.085		0.004		<10
200	0.633	0.410	0.571	0.573	0.263	0.252	0.290	0.305	0.054	0.057	pos	0.063	0.064	<3,8		neg	0.093		0.004		<10
65	0.408	0.390	0.399	0.394	0.338	0.364	0.589	0.633	0.056	0.051	pos	0.048	0.048	<3,8		neg	0.096		0.006		<10
127	0.337	0.318	0.251	0.232	0.342	0.321	0.218	0.157	0.052	0.056	pos	0.057	0.056	<3,8		neg	0.094		0.002		<10
148	0.179	0.122	0.119	0.120	0.280	0.269	0.292	0.302	0.057	0.059	pos	0.059	0.058	<3,8		neg	0.109		0.005		<10
C1	0.674	0.654	0.674	0.654	0.744	0.786	1.208	1.263	1.308	1.448	pos	0.335	0.325	12.30	12.40	neg	7.370	9.440	2.335		15
C2	0.232	0.214	0.232	0.214	0.326	0.342	0.945	0.917	0.855	0.989	pos	0.151	0.144	5.56	5.74	neg	1.460	1.840	0.096		<10
C3	0.157	0.159	0.183	0.218	0.872	0.838	3.866	>4,0	1.443	1.661	pos	1.418	1.386	73.60		pos	1.970		3.500		40
C4	3.979	OVRFLW 80		3.979	OVRFLW		1.728	1.607	>4,0	>4,0	3.511	3.538	pos	1.404	1.386	>400		pos	90.800		3.282
C5	OVRFLW 25.500		OVRFLW 3.475		OVRFLW 60		OVRFLW		1.307	1.275	3.173	3.154	2.981	3.021	pos	0.157	0.155	44.80		neg	

IMD ImmunoDiagnostics, HongKong; EDI, Epitope Diagnostics Inc., San Diego, CA; VIR, Vircell, Granada, Spain; IDV, IDvet, Grabels, France; WB, Western Blot; EUR, Euroimmun Medizinische Labordiagnostik AG, Lübeck, Germany; LIA, Liaison, DiaSorin S.p.A, Saluggia, Italy; ROC, Roche Diagnostics Deutschland G.m.b.H, Mannheim, Germany; WAN, Beijing Wantai Biological Pharmacy Enterprise Co., Ltd., Beijing, China; TA, total antibody; N, SARS-CoV-2 nucleocapsid protein; S, SARS-CoV-2 spike glycoprotein; BL, baseline; FU, follow-up; neg, negative; pos, positive; OVRFLW, overflow.

*, numbers 1 and 2 indicate raw data of duplicates.
